# Synovialosarcome de l’avant-bras droit

**DOI:** 10.11604/pamj.2018.30.124.15900

**Published:** 2018-06-13

**Authors:** Youssef Zemmez, Adnane Arrami

**Affiliations:** 1 Service de Dermatologie, Hôpital Militaire d'Instruction Mohammed V, Rabat, Maroc; 2 Service de Radiologie, Hôpital Militaire d'Instruction Mohammed V, Rabat, Maroc

**Keywords:** Synovialosarcome, histlogie, chirurgie, Synovial sarcome, histlogy, surgery

## Abstract

We report the case of a 60-year old female patient, followed up for hypertensive heart disease for 04 years, presenting to the Dermatological Department with a mass in the right forearm which had evolved and gradually increased in size over the previous year. Clinical examination showed soft rounded, voluminous painless, tumor-like crusted ulcerative lesion measuring 8 cm in large-diameter, fixed with respect to both planes, at the level of the mid-parts of the posterolateral area of the right forearm (A). Standard X-ray showed homogeneous opaque mass with sparing of the opposite cortex (B). Magnetic resonance imaging showed voluminous multicompartmentalized cystic and tissue cutaneous-subcutaneous process infiltrating the subcutaneous grease and in contact with extensor muscles (C). Histology revealed poorly differentiated sarcomatous pleomorphic cells proliferation (D). Immunohistochemical study confirmed the diagnosis of synovial sarcoma. The patient was referred to the Division of Plastic Surgery for surgical treatment.

## Image en médecine

Patiente âgée de 60 ans, suivis pour cardiomyopathie hypertensive depuis 04 ans, qui a consulté en dermatologie pour une masse de l’avant-bras droit évoluant depuis un an et augmentant progressivement de taille. L’examen clinique a objectivé une lésion tumorale molle, arrondie, volumineuse mesurant 8 centimètres au grand diamètre, indolore, fixe par rapport aux deux plans, à surface ulcéro-crouteuse et siégeant au niveau du tiers moyens de la face postéro-latérale de l’avant-bras droit (A). La radiographie standard a objectivé une masse opaque homogène respectant la corticale en regard (B). L’Imagerie par résonnance magnétique était en faveur d’un volumineux processus cutanéo-sous cutané à double composante tissulaire et kystique multi-cloisonnée infiltrant la graisse sous cutanée et arrivant en contact intime avec les muscles extenseurs (C). L’histologie a objectivé une prolifération sarcomateuse peu différenciée à cellules pléomorphes (D) complétée par une étude Immuno-histochimique qui a confirmé le diagnostic d’un Synovialo-sarcome. La patiente a été adressée en chirurgie plastique pour bénéficier d’un traitement chirurgical.

**Figure 1:**
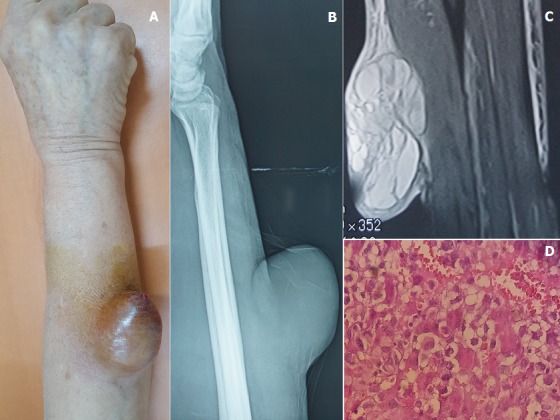
A) masse tumorale de l’avant-bras droit; B) masse opaque homogène respectant la corticale en regard; C) processus cutanéo-sous cutané à double composante tissulaire et kystique multi-cloisonnée; D) histologie: prolifération sarcomateuse peu différenciée à cellules pléomorphes

